# Stress-Induced Changes in Alternative Splicing Landscape in Rice: Functional Significance of Splice Isoforms in Stress Tolerance

**DOI:** 10.3390/biology10040309

**Published:** 2021-04-08

**Authors:** Showkat Ahmad Ganie, Anireddy S. N. Reddy

**Affiliations:** 1 Department of Biotechnology, Visva-Bharati, Santiniketan 731235, WB, India; 2 Department of Biology and Program in Cell and Molecular Biology, Colorado State University, Fort Collins, CO 80523, USA

**Keywords:** Alternative splicing, abiotic stress, biotic stress, *Oryza sativa*, splicing factor, virulence

## Abstract

**Simple Summary:**

Environmental stresses adversely affect rice production. Understanding the molecular responses of rice to these stresses will have an enormous impact on the sustainable production of this economically important food crop. Alternative splicing (AS) is a vital post-transcriptional modulator of gene expression that amplifies the proteome diversity and regulates many physiological processes essential for mounting responses to stresses in plants. Recent studies in rice have revealed that AS is significantly altered in response to diverse abiotic and biotic stresses to rapidly reprogram gene expression that is optimal for plant survival under these unfavorable growth conditions. We review the current understanding of how AS controls the responses of rice to environmental stresses. We also summarize the different molecular entities targeted by AS under stress conditions, such as abiotic stress-responsive genes, and *trans*-acting splicing factors that modulate AS. Moreover, to gain insights into sustainable pest control, we also discuss the role of AS in the growth, development, and virulence of rice pathogens. Collectively, this information could provide insights into the roles of AS in rice response to environmental stresses, and potentially developing stress-resilient rice cultivars.

**Abstract:**

Improvements in yield and quality of rice are crucial for global food security. However, global rice production is substantially hindered by various biotic and abiotic stresses. Making further improvements in rice yield is a major challenge to the rice research community, which can be accomplished through developing abiotic stress-resilient rice varieties and engineering durable agrochemical-independent pathogen resistance in high-yielding elite rice varieties. This, in turn, needs increased understanding of the mechanisms by which stresses affect rice growth and development. Alternative splicing (AS), a post-transcriptional gene regulatory mechanism, allows rapid changes in the transcriptome and can generate novel regulatory mechanisms to confer plasticity to plant growth and development. Mounting evidence indicates that AS has a prominent role in regulating rice growth and development under stress conditions. Several regulatory and structural genes and splicing factors of rice undergo different types of stress-induced AS events, and the functional significance of some of them in stress tolerance has been defined. Both rice and its pathogens use this complex regulatory mechanism to devise strategies against each other. This review covers the current understanding and evidence for the involvement of AS in biotic and abiotic stress-responsive genes, and its relevance to rice growth and development. Furthermore, we discuss implications of AS for the virulence of different rice pathogens and highlight the areas of further research and potential future avenues to develop climate-smart and disease-resistant rice varieties.

## 1. Introduction

Rice is acclaimed as the most economically important crop serving as a staple food for human consumption worldwide. The rapidly growing global population has profoundly increased the demand for rice, especially in Asian countries [1]. According to the latest report of FAO, global rice production in 2019 was 502.8 million tons (mt); however, the global rice demand in 2020/21 is expected to increase to a record level of 514 mt [2]. Ensuring sufficient rice supply to the growing population has remained a serious challenge for rice breeders, which is exacerbated by the adverse environmental conditions. Environmental stresses severely affect different facets of growth and development and eventually reduce the productivity and yield of rice [3,4]. Therefore, the adverse environmental factors together with the increasing population compel us to develop next-generation climate-smart rice cultivars for sustainable agriculture. However, accomplishing this requires a comprehensive understanding of the complex regulatory mechanisms that contribute to stress tolerance in rice.

Plants have evolved different sophisticated regulatory strategies for adapting to biotic and abiotic stresses [5,6]. Regulation of gene expression at the transcriptional, post-transcriptional, and post-translational levels is crucial for the survival of plants under these stresses [5,7,8]. In recent years, post-transcriptional gene regulation has been increasingly recognized as an important regulatory mechanism in plant growth, development, and especially in stress responses. AS is a pervasive and highly dynamic post-transcriptional regulatory process, which plays a pivotal role in reprogramming gene expression in plants in responses to environmental stresses [9,10], and is gaining attention in the quest to develop stress-tolerant plants. AS enables regulated production of multiple distinct mRNAs and protein variants from a single gene via differential joining or skipping of exons or portions of exons and removal of introns within a pre-mRNA transcript [11]. For general mechanisms of AS, the reader is referred to recent reviews [12,13,14]. Five major types of AS events include intron retention (IR), exon skipping (ES), alternative 5′ splice sites (A5SS; alternative donor site), alternative 3′ splice sites (A3SS; alternative acceptor site), and mutually exclusive exons (MXE) [15,16]. The IR is the most common type of AS event in plants, whereas the ES is the most prevalent in animals [17,18]. The difference in prevalent AS events between plants and animals is likely due to the architectural differences in plant and animal genes, such as the presence of much shorter introns in plants, and also due to the intron-defined splicing process in plants [11,18]. Transcripts with the retained introns are present in polysomes indicating their export to the cytoplasm, and potential regulatory roles in plants [17,19]. Intriguingly, alternative transcripts with retained introns tend to be from genes that function in diverse stress responses [17].

AS is a highly regulated process that requires *cis*-elements (located in both exonic and intronic sequences of pre-mRNA) and *trans*-acting factors that bind to these *cis*-elements—a basic mechanism that is quite conserved in eukaryotes [20,21]. An interplay between these regulatory elements (exonic/intronic splicing enhancers and silencers) and *trans*-factors determines the fate of a particular exon or intron be retained or excluded from the mature transcript by either facilitating or hampering the assembly of the spliceosome [20,22]. The canonical mechanism of AS suggests that serine/arginine-rich (SR) proteins and heterogeneous nuclear ribonucleoproteins (hnRNPs) constitute the important *trans*-factors that bind splicing enhancers and silencers to facilitate and suppress splicing, respectively [16]. SR proteins play crucial roles in both constitutive and AS and regulate the recruitment of splicing machinery to splice sites by mediating the protein-protein interactions among other splicing factors [23].

Besides, increasing evidence indicates that AS is also regulated by the state of chromatin in plants [24]. Due to the co-transcriptional regulation of splicing, it is influenced by the elongation rate of RNA polymerase II, which is in turn controlled by chromatin structure [25]. In fact, the IR events are highly enriched in those genomic regions of plants where chromatin is highly accessible [25]. DNA methylation also regulates AS in rice by affecting the dynamic chromatin structure [26]. The epigenetic regulation of AS is also involved in plant stress responses. For example, modulation of AS through histone methylation serves as a key mechanism for temperature sensing in Arabidopsis [27]. Likewise, the chromatin remodeler ZmCHB101 affects the AS contexts in maize by altering the chromatin and histone modification status, as well as the elongation rate of RNA polymerase II in response to osmotic stress [28]. Moreover, noncoding RNAs have also emerged as key regulators of AS patterns in plants, which, in turn, influence the miRNA-mediated regulation of gene expression associated with different biological processes [29,30,31,32,33,34]. Together, these findings reveal an additional epigenetic layer in regulating AS in plants. 

AS is a functional innovation of eukaryotic genomes as it increases the proteome complexity and functional capacity in a cell, and hence, the phenotypic diversity [20,35]. AS of pre-mRNAs has various molecular consequences at the mRNA and protein levels (Reviewed by Laloum et al. [9]). Depending on the different AS events and the alternative inclusion or exclusion of fragments in the mature transcript, AS can produce protein isoforms with distinct domains that may carry different molecular functions. AS-mediated alteration of the open reading frame (ORF) often results in the generation of truncated proteins, due to the introduction of a premature termination codon (PTC) in the mature transcript. The PTC-containing transcripts are then recognized by specific cellular proteins, which mediate the elimination of these transcripts through nonsense-mediated decay (NMD)—a cytoplasmic translation-coupled mechanism that degrades PTC-containing mRNAs [36,37]. Several reports also suggest that PTC-containing mRNAs escape NMD to form truncated translation products with regulatory functions, such as modification of protein interaction networks, negative regulation of protein dimerization, and alteration of post-translational modifications [38]. The truncated isoforms can also act in autoregulatory loops to control the gene transcription [39]. It is also suggested that the production of these truncated proteins serves as a potential way of fine-tuning the amount of functional protein in the cell [40]. AS also occurs in the untranslated regions (UTRs) of pre-mRNAs. The AS in 5′-UTRs is suggested to influence translation efficiency by generating transcript variants with altered upstream ORFs (uORFs) or riboswitches, whereas AS in the 3′-UTR can affect miRNA binding sites in the transcript variants [41].

AS patterns are highly altered in response to different environmental stresses, which allows plants to rapidly adjust the abundance and function of crucial stress-responsive proteins [9,10]. Comparative plant genome analysis has shown that genes undergoing AS have a greater evolutionary rate, which may be associated with better adaptation to adverse environmental conditions [38]. Many next-generation sequencing (NGS) studies have revealed that a considerable number of AS events occur in plants in response to environmental stresses [10,42]. Global transcriptomic studies using RNA-seq have revealed that stress-induced AS occurs mainly in regulatory genes, such as transcription factors, protein kinases, and splicing factors [10,42,43]. Because most of the stress-induced AS patterns are discovered using transcriptomic methods, the functional significance of a majority of the stress-regulated AS events in plants is yet to be demonstrated. However, increasing evidence reveals that AS in individual genes plays critical functional roles in various aspects of plant stress responses [9,10,44,45]. Besides, emerging evidence indicates that AS is involved in an intriguing adaptive response of stress-memory in plants. Heat stress priming-induced AS memory has been observed in Arabidopsis plants, which helps them survive subsequent lethal stress by generating appropriate stress-responsive splice isoforms [46].

As in other plants, AS is a key regulatory mechanism that is modulated in response to various biotic and abiotic stresses in rice also. As discussed below, RNA-seq has been widely used in rice to analyze AS in response to various abiotic (including drought, salinity, extreme temperatures, light stress, hypoxia, metal stress, mineral stress, and abscisic acid) and biotic stresses (including bacteria, fungi, insects). In addition to the transcriptomic studies, there are several studies on AS in individual candidate genes in rice with functional significance for stress tolerance. In this review, we present a comprehensive overview of AS landscape of rice under different abiotic and biotic stresses. We also review different molecular entities targeted by AS under stress conditions, such as stress-responsive genes and *trans*-acting splicing factors that modulate AS. Further, to gain insights into sustainable pest control, we also discuss the role of AS in the growth, development, and virulence of rice pathogens.

## 2. Abiotic Stress

### 2.1. Transcriptomic Analyses of Abiotic Stress-Induced AS

High-throughput sequencing technologies have become indispensable to study epigenomics, genomics, and transcriptomics. In particular, RNA-seq is a powerful tool that has truly revolutionized our ability to analyze plant transcriptomes, especially as it relates to environmental stresses. RNA-seq has catalyzed plant science research by enabling the rapid profiling and detailed investigation of transcriptional and post-transcriptional processes in any plant with or without a sequenced genome [24,47]. RNA-seq has rapidly superseded other transcriptomic methods for studying gene expression dynamics as it offers many advantages, such as detection of transcripts beyond the limit of microarrays, increased dynamic range for more extreme limits of detection, and the ability to identify novel, rare, low-abundance, and alternatively spliced transcripts in a single sequencing run [24,47,48]. It can detect not only the differential gene expression, but also the differential AS events in different genetic backgrounds under a particular condition [11]. Besides, RNA-seq can robustly detect single nucleotides polymorphisms (SNPs) [49].

RNA-seq studies in rice have provided new perspectives on unknown transcript dynamics and led to significant progress in identifying genes and molecular mechanisms that regulate important traits governing abiotic stress tolerance [50,51,52,53,54,55]. These studies have revealed altered patterns of splicing in key genes associated with various abiotic stress responses. Below, we discuss rice transcriptome studies that are aimed at understanding AS in rice in response to various abiotic stress conditions.

#### 2.1.1. Relationship between Stress-Induced AS and Stress-Tolerance

The transcriptome sequencing is performed on the genotypes with inherent contrasting genetic responses to stresses. This is done to unravel the key molecular components that confer differential stress tolerance in the contrasting genotypes. Similarly, RNA-seq has provided novel insights into differential AS in contrasting rice genotypes under different abiotic stresses, which might offer new avenues for rice breeding. A transcriptomic study revealed divergence in drought-induced AS patterns between upland drought-tolerant and lowland drought-sensitive rice cultivars, with more cultivar-specific AS events in tolerant than sensitive cultivar [56]. Spliceosome-associated genes encoding hnRNP G, SR, and RNA helicase proteins were largely subjected to AS in upland cultivar, and this distinctive AS in spliceosomal proteins may lead to the differential AS in the other genes. In fact, genes associated with stress-associated pathways, such as circadian rhythm and oxidative phosphorylation, showed AS patterns specifically in upland rice. Interestingly, several genes encoding spliceosomal proteins with different AS events were located in drought-responsive quantitative trait loci (QTLs), implicating a role for these genes in rice response to drought. These results indicate that AS of pre-mRNAs encoding splicing factors can be a crucial strategy adopted by upland rice during long domestication to tolerate drought. Another transcriptomic study associated with salt stress in contrasting rice genotypes has revealed down-regulation of AS, specifically in sensitive genotype, which might contribute to its susceptibility to salt stress [54]. A systematic comparison of transcriptomic profiles of contrasting rice genotypes under salt stress has uncovered AS to be a crucial mechanism underlying salt tolerance in salt-tolerant genotype FL478 [57]. Although more AS events were identified in susceptible genotype than the tolerant, AS events in genes involved in crucial salt tolerance-associated processes of ion-transport and signaling pathways were more frequent in tolerant genotype. These alternatively spliced variants were either highly expressed (*bZIP*, *PP2C* variants) or specifically expressed (*WRKY30*, *MAPK kinase*, and *HAK25*, *ABC* and *ZIFL* transporter variants) in the tolerant genotype [57]. Similar results were reported in the case of salt-tolerant barley and susceptible rice genotypes under salt stress. Although more AS events were found in rice than barley, a higher level of AS in the barley genes related to ion-transporters and transcription factors contribute to the higher salt tolerance in barley, due to the superior K^+^/Na^+^ homeostasis [58]. Moreover, one of the SR splicing factors was specifically expressed in barley. Likewise, a separate transcriptomic analysis revealed higher AS events in salt-sensitive than salt-tolerant genotype, but a higher number of alternatively spliced isoforms were specifically expressed in tolerant genotype as compared to sensitive genotype under salt stress [59]. In this study, although most of the AS events decreased in all the analyzed rice genotypes, including a drought-tolerant genotype under desiccation stress, high expression of some spliced transcripts was observed exclusively in the drought-tolerant genotype, which might contribute to drought tolerance. Alkali stress response in contrasting rice cultivars revealed more AS events in alkali sensitive cultivar; however, some alkali-responsive genes were differentially alternatively spliced between the two genotypes, suggesting a potential role for AS in the differential response of the two cultivars to alkali stress [52]. In another study, a lower number of AS events were found in an iron stress-tolerant rice genotype under iron-excess stress than control [60]. However, among the different splicing events, IR was more prevalent, and the splicing events were altered in an organ-specific manner under stress.

Collectively, the above five studies indicate that higher stress-induced AS in a genotype does not necessarily correlate with stress tolerance, but the frequency of genotype-specific events, the abundance of AS transcripts related to key stress-responsive processes, and the nature of splicing events are likely to contribute to stress tolerance. It is possible that the bulk of alternatively spliced transcripts may be subjected to degradation by NMD and may not be translated. In fact, only about 14% of the total alternatively spliced transcripts are translated into peptides under hypoxic stress in rice [61]. In a related study, treatment with a salt-tolerant endophyte was shown to confer salt stress adaptation and decrease the AS in salt-sensitive rice plants as compared to their untreated counterparts [62]. It was proposed that treatment with the endophyte causes a lowered perception of salt stress signaling, which might not activate the stress-responsive mechanism of AS in the treated plants, underscoring the importance of AS in response to stress. Another RNA-seq analysis at the reproductive stage of a drought-resistant rice maintainer line and its recurrent parent has revealed a similar level of gene expression at the transcriptional level under drought; however, the increased level of AS in transcription regulatory networks likely contributes to the genetic basis of adaptation to drought in the maintainer line [51]. It was reported that differentially expressed genes (DEGs) involved in the biological process of chloroplast components undergo increased AS in a maintainer line and are regulated by six transcription factor families, adding an extra regulatory level to genetic improvement of drought resistance in this line. The higher drought resistance in offspring could be due to AS-mediated limited damage to the photosynthetic apparatus in chloroplasts for maintaining the energy metabolism under drought. Li et al. [63] reported that changes at the transcriptional level do not always correspond to the changes in protein abundance. They have shown that in salt-tolerant genotype, the number of up-regulated DEGs was markedly higher at the translational level as compared to transcriptional level, whereas the number of up-regulated DEGs in susceptible genotype was comparable at the two levels. However, the number of up-regulated DEGs in the susceptible genotype was lower at the translational level as compared to salt-tolerant genotype, which could be attributed to the almost 3-fold abundance of alternatively spliced transcripts in tolerant genotype as compared to susceptible genotype that could increase proteome diversity in tolerant genotype under salt stress.

#### 2.1.2. AS-Mediated Spatial Regulation of Stress-Responses

Rice responds to abiotic stresses by spatially activating distinct molecular processes [64,65]. Transcriptomic analyses have revealed that AS may play key roles in regulating the stress-induced processes in roots and shoots of rice. A high level of AS occurs in root and shoot of rice under aerobic conditions [53], with more splicing events occurring in the aerobically-induced chloroplast development-associated genes (coding for tetratricopeptide repeat domain-containing protein and GOLDEN2-LIKE1 transcription factor) in the shoots; and higher splicing events taking place in the aerobically-induced plastid-related gene (coding for PAP fibrillin domain-containing protein) in the roots. Similarly, in wild rice, *Oryza longistaminata*, the number of genes undergoing AS and the frequency of splicing events greatly increase in shoots and rhizomes under chilling stress. In shoots, AS occurs exclusively in the chilling-induced genes associated with regulation of gene expression and photosynthesis; whereas, in rhizomes, the chilling-induced genes associated with Ca^2+^-binding undergo AS [66]. It indicates that in response to chilling, AS may regulate calcium-mediated signal transduction in rhizomes, whereas it may play crucial roles in regulating transcription factors having diverse functions and also the photosynthesis in shoots. Therefore, organ-specific AS may contribute to the regulation of long-term chilling tolerance in *O. longistaminata*. In both of these studies, a higher number of AS events were observed in the shoots than in the roots, and vice-versa for the number of DEGs. In response to stress, the DEGs involved mostly in signaling pathways (including hormone signaling) are activated in roots, which may send stress signals to shoots where they may induce higher AS of certain genes for stress adaptation by regulating the expression of AS-related factors. Wild rice species, which are generally tolerant to various environmental stresses, appear to reap the benefits of AS for abiotic stress tolerance differently. Under phosphorus stress, *O. rufipogon* adopts a distinct AS-based strategy than the one used by *O. longistaminata* under cold stress. In *O. rufipogon*, phosphorus stress causes up-regulation of Lsm8 and U1A spliceosome-related proteins and down-regulation of SPF and SR splicing factors [67]. It was proposed that up-regulation of Lsm8 and U1A may increase the AS, while the down-regulation of the two splicing factors may lead to the changes in pre-mRNA splice sites under phosphorus stress, which may be associated with the phosphorus stress tolerance in this wild rice species. The use of increased AS by *O. rufipogon* as a strategy to deal with the phosphorus stress was also supported by the finding that phosphorus stress reduces the level of transcription-related genes and increases the RNA splicing and translation elongation, indicating that phosphorus-induced gene expression operates at the post-transcriptional level.

#### 2.1.3. Stress-Induced Changes in AS Represent an Independent Layer of Gene Regulation

In addition to the DEGs, AS also occurs in genes that are not affected at the transcription level between the samples (called differentially alternatively spliced genes; DASGs) under stress. In other words, AS can also be independent of transcriptional regulation, which indicates that AS serves as an additional layer of regulation [11]. In this regard, almost 1741 DAS events were identified in rice seeds under hypoxia, out of which, more than 95% did not overlap with DEGs, suggesting that DASGs is a different group of genes than that of DEGs that are responsive to hypoxic germination, suggesting that AS may have a distinct role in the germination of rice seeds under hypoxia [61]. Intriguingly, the DEGs and DASGs are functionally enriched in distinct cellular processes and metabolic pathways, with cellular metabolism and cell growth mainly enriched in DEG data; while protein degradation, post-transcriptional regulation, and transport processes mainly enriched in DASG data sets. This study also found that just 38% of hypoxia-induced DAS events were translated into peptides in the germinating seeds, indicating that most of the DAS events may be degraded via NMD. Similar results for the nonoverlap between DEGs and DASGs and their involvement in functionally distinct processes have been obtained in rice under mineral stress [11], drought [68], and cadmium stress [50]. It is inferred from these studies that regulation of stress-induced gene expression at transcriptional and post-transcriptional (AS) levels is independent, supporting the notion that environmental stresses influence the gene expression at transcriptional and AS levels [21]. These two mechanisms act in concert to promote the performance of rice under stress conditions; e.g., high transcriptional expression of cellular metabolism-related genes can lead to significant misfolded protein production under hypoxia, whereas the AS-mediated generation of new isoforms may potentially degrade these misfolded proteins to alleviate the hypoxic stress [61]. This suggests that adaptation of rice to stresses may have been shaped by distinct transcriptional rewiring of AS, as well as by gene expression variation. 

The transcriptomic studies also reveal that SR splicing factors themselves undergo extensive DAS, without exhibiting differential expression, under abiotic stresses [11,50,61] as a means to regulate the AS of more diverse downstream genes. In fact, Chen et al. [61] report that hypoxia-induced AS in SR proteins can generate diverse isoforms of these splicing factors with a different choice of splice sites, and this was associated with the abundance and preferential usage of certain noncanonical 5′-splice sites for germination under hypoxia.

### 2.2. Abiotic Stress-Induced AS of Candidate Genes

#### 2.2.1. Abiotic Stress-Responsive Genes

**Figure 1:**
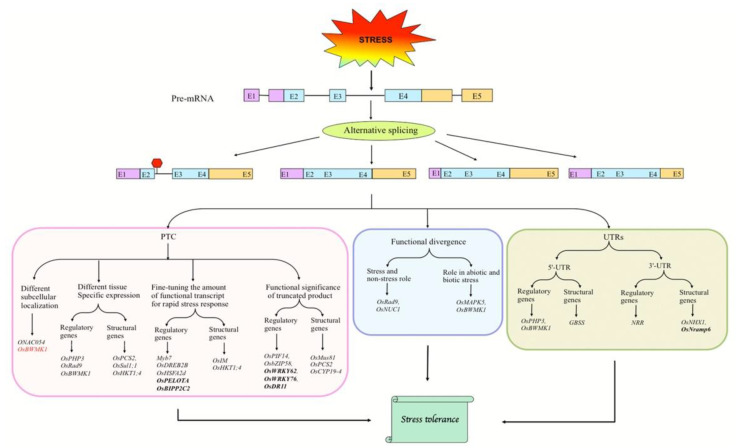
Various modes of AS-dependent regulation of splice isoforms in response to biotic and abiotic stresses. Under stress conditions, the pre-mRNA of stress-responsive genes may generate alternative transcripts with PTC that may regulate certain features of the splice isoforms, such as subcellular localization and spatial expression, which may fine-tune the level of functional protein isoforms or produce proteins with altered functions (**Left**). The alternative transcripts may be functionally distinct (**Middle**). Besides the coding region, stress-induced AS in UTRs of pre-mRNAs can also regulate splice isoforms (**Right**). Genes in bold represent biotic stress-responsive genes, and the remaining ones are abiotic stress-responsive. Gene shown in red functions in both biotic and abiotic stresses. Boxes and lines represent exons and introns, respectively. In transcripts, purple, sky blue, and orange colors represent 5′-UTR, coding region, and 3′-UTR, respectively. PTC, premature termination codon; Red hexagon represents PTC.

Although global transcriptome analyses have identified plenty of abiotic stress-induced alternatively spliced transcripts, the functional significance of only some of them in adaptive stress responses has been demonstrated. The protein-coding genes can be either structural or regulatory, with the proteins encoded by structural genes having structural or functional properties in the cell—while those encoded by regulatory genes regulate the expression of structural genes [69]. Based on our extensive literature survey, we found that rice genes encoding regulatory proteins are more likely to undergo AS under various abiotic stresses, and more importantly, the functional significance of AS events in these genes has been analyzed (Figure 1; Table S1 [70,71,72,73,74,75,76,77,78,79,80,81,82,83,84,85,86,87,88,89,90,91,92,93,94,95]). It follows that AS is an elegant and energy-saving mechanism particularly targeting those genes which can, in turn, regulate a diverse set of downstream structural genes for activating the adaptive responses to stresses. However, some structural genes of rice also undergo stress-induced AS, which has important implications for rice abiotic stress tolerance. AS in abiotic-stress-responsive regulatory and structural genes of rice acts through diverse mechanisms as illustrated below.

One of the most common mechanisms by which AS can regulate the transcript abundance in response to stress is via the cytoplasmic RNA degradation system of NMD. Transcripts that are particularly prone to NMD have classical features of retained introns, PTC, uORFs, etc. [15]. However, these transcripts may also escape NMD to form truncated proteins with key regulatory functions under stress [9,20,38].

##### Fine-Tuning the Abundance of Functional Transcripts

**Figure 2:**
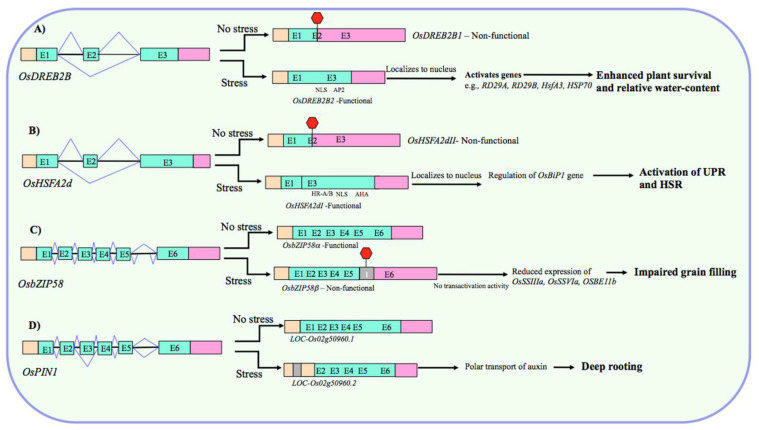
Representative examples of AS-mediated regulation of gene expression in rice under heat/drought stress conditions. (**A**) Under nonstress conditions, nonfunctional (due to frameshift and PTC) form predominates, while the functional form (with intact NLS and typical AP2/ERF domain) is produced under drought to increase drought tolerance potentially by enhancing the expression of stress-related genes [70]. (**B**) Under nonstress conditions, nonfunctional (due to PTC) form predominates, while the functional form (with intact DNA-binding-, oligomerization-, and activation-domains) is produced under heat stress which participated in heat stress response (HSR) by regulating the expression of unfolded protein response (UPR) marker gene *OsBip1* [72]. (**C**) Under nonstress conditions, functional form predominates, while the nonfunctional form (due to IR-mediated PTC), lacking bZIP domain, is produced predominantly under high temperature, which causes impaired grain filling by repressing the expression of genes associated with grain filling [76]. (**D**) An IR event in the *OsPIN1* transcript has a potential functional significance in regulating the rice deep rooting under drought by facilitating the polar auxin transport [68]. Boxes and lines represent exons and introns, respectively. In transcripts, peach, aquamarine, and violet colors represent 5′-UTR, coding region, and 3′-UTR, respectively. Red hexagons represent PTC. “I” in the splice isoforms indicates an intron retention event.

By producing truncated nonfunctional proteins, AS through timely IR or PTC serves as a potential way of fine-tuning the amount of stress-responsive functional protein in the cell during abiotic stress. For example, drought and heat stress-induced AS regulates the cellular levels of *OsDREB2B*, which has a nonfunctional (*OsDREB2B1*) and a functional transcript form (*OsDREB2B2*). The nonfunctional and functional forms are predominant under non-stress and stress conditions, respectively. Under drought, the functional form operates to increase drought tolerance by enhancing the plant survival rate and relative water content (Figure 2A) [70]. Similarly, hypoxia-induced *myb7* has an unspliced (with two retained introns) and a spliced form, and the ratio of these isoforms changes under aerobic and hypoxic conditions. The spliced form increases the expression of *myb*-related genes in hypoxic roots [71]. Likewise, the heat shock factor gene *OsHSFA2d* constitutively produces the inactive forms (*OsHSFA2dII* and *OsHSFA2dIII*) under non-stress conditions, whereas under heat stress, it generates the transcriptionally active form (*OsHSFA2dI*), which participates in heat stress response by regulating the unfolded protein response (UPR) (Figure 2B) [72].

Through IR and PTC, AS also regulates the abundance of functional stress-responsive transcripts of some important rice structural genes. The best example of this is provided by the OsHKT1;4-mediated Na^+^ exclusion from the leaf blades of salt-stressed rice [73]. *OsHKT1;4* has three transcript variants, among which, only the canonically spliced one is functional. The high Na^+^ excluding pokkali is superior to salt-sensitive Nipponbare in minimizing the Na^+^ load in the young photosynthetic leaf blades by maintaining a high ratio of functional *OsHKT1;4* transcript variants in the corresponding sheaths. Similarly, the pre-mRNA of an oxidase gene *OsIM* produces functional (*OsIM1*) and pseudo-transcript (*OsIM2*), and both exist under non-stressed conditions; however, under salt stress, the *OsIM1*/*OsIM2* ratio greatly increases because of the significantly reduced *OsIM2* levels [74], that may relieve salinity-induced oxidative stress. Likewise, the iron-superoxide dismutase gene *OsFe-SOD* has two isoforms, *OsFe-SODa* and intron-containing *OsFe-SODb*, which respectively show significantly high and low expression under cold stress [96].

Overall, these studies indicate that an inactive transcript variant may exist to keep its gene constitutively active without affecting the plant growth, while the onset of stress may send a signal to the splicing apparatus to alter the splicing pattern to produce the active stress-responsive form. The rationale behind this elegant ON/OFF switch is that plants can promptly generate the active transcript forms as an adaptive strategy when subjected to abiotic stress, and by doing so, they save time that is otherwise needed for transcription and transcript accumulation of the gene under discussion [70].

##### Regulatory Role of IR in Stress Responses

Previously, limited attention was paid to IR primarily because it was assumed to be a product of missplicing with no physiological implications, but diminishing the gene expression by NMD. However, accumulating evidence has revealed that it acts as a critical component of the controlled gene expression program and plant abiotic stress responses [45]. The case studies of individual IR events have demonstrated it as an exemplar of regulated splicing and have provided key mechanistic insights into regulating abiotic stress responses in rice. For example, cold-induced AS in the pre-mRNA of phytochrome-interacting factor *OsPIF14*, which regulates cold tolerance in rice by repressing the expression of *OsDREB1B*, introduces a PTC via intron-retention, which leads to the production of a transcriptionally inactive truncated protein lacking a complete bHLH domain [75]. At severe temperature, the inactive form *OsPIF14β* is up-regulated and the active form *OsPIF14α* is down-regulated. This has a functional significance because *OsDREB2B* is crucial for rice cold tolerance and its activation (due to down-/up-regulation of α-/*β*-form) at lower temperature leads to the accumulation of OsDREB2B, and subsequent cold tolerance. The IR in another transcription factor gene *OsbZIP58* has an important role in mediating the effect of high temperature on rice grain filling and seed quality control [76]. The high temperature triggers AS of *OsbZIP58* to generate mainly the intron-retaining truncated form *OsbZIP58β* over the full-length form *OsbZIP58α* (Figure 2C). The predominance of transcriptionally less active *OsbZIP58β* at high temperature impairs the accumulation of storage materials in rice grains possibly by diminishing the amount of functional OsbZIP58 protein, leading to the reduced *trans*-activation of genes associated with the grain filling, such as *OsSSIIIa*, *OsSSVIa*, *OsBEIIb*, *GBSSI*, and *OsBEI* (Figure 2C). Intriguingly, this IR event is also associated with the heat-sensitivity of contrasting rice genotypes [76], suggesting that it can potentially act as a heat tolerance marker in rice. Another example of a functional AS event comes from an auxin efflux carrier gene *OsPIN1* [68]. Dynamic AS is associated with deep rooting-mediated drought avoidance in rice and overexpression of *OsPIN1* containing a retained intron improves the ratio of deep rooting (RDR) in rice (Figure 2D). Because *OsPIN1* gene and the spliced isoform did not show any differential expression between low and high RDR rice genotypes and because this IR event was highly up-regulated in deep roots of high RDR genotypes only, it is likely that IR event in *OsPIN1* is important for rice root development and increases RDR apparently by facilitating the auxin transport [68]. In yet another case, the unspliced form of rice *myb7* having two retained introns and a short leucine zipper is predominant in the aerobic organs; while the introns are spliced out under hypoxic, suggesting a regulatory role of this intron as an efficient switch to alternatively express myb regulatory protein under contrasting conditions [71]. Alternatively, if we analyze this finding from the perspective of aerobic conditions being stressful to rice [47], then this IR as a functional event makes sense because the leucine zipper motif encoded by unspliced intron can potentially interact with the myb factor [97] to activate the transcription of the target genes under aerobic stresses.

Small sequence variations in certain genes can have a great impact on their AS and expression. For example, insertion of a mutation in the post-translational modification-related glycosylphosphatidylinositol gene *OsGPI8* causes IR, PTC, and loss of function, especially at elevated temperature [98]. The IR-mediated silencing of *OsGPI8* impairs cell wall biosynthesis, cell division, and cell shape in rice plants, leading to their fragility and drooping of shoots. This finding indicates a potential interplay between the processes of post-transcriptional and post-translational gene regulation for eliciting the proper abiotic stress responses in rice.

Analyses of IR in structural genes have also revealed prominent functional roles in biological settings for rice abiotic stress tolerance. The DNA repair gene *OsMus81* produces two splice variants (the main *OsMus81α* and the truncated *OsMus81β*), which are up-regulated under intense light, UV-C, and γ-radiation [77]. Because OsMus81β lacks the helix-hairpin-helix motif, only OsMus81α interacts with Mms4/Eme1 to form a hetero-trimeric endonuclease complex, which functions in nucleotide excision repair. Besides, both of them interact with recombination repair protein OsRad54.However, because OsRad54-OsMus81β interaction is stronger than that of OsRad54-OsMus81α, OsMus81β may operate at the later stages of homologous recombination pathway for DNA repair [77]. This indicates that these alternative transcripts may repair the DNA damage by different repair mechanisms. Similarly, the phytochelatin synthase gene *OsPCS2* has a canonically spliced variant (*OsPCS2a*) and the alternatively spliced PTC-containing variant (*OsPCS2b*). These isoforms are differentially expressed in cadmium-stressed roots and shoots. It was suggested that the unproductive *OsPCS2b* splice variant has a regulatory role in controlling the tissue-specific expression of the functional variant during cadmium stress [99]. Further, *Oryza* species-specific sequence distinctions in the vacuolar NHX-type antiporter (*OsNHX1*) relate to the differential intron-retention at the 5′ and 3′ ends of its transcript [100]. The recent IR event in 5′-UTR is found only in AA *Oryza* genomes, while the retention of the 13^th^ intron is more ancient in origin and also occurs in *OcNHX1* of halophytic wild rice (*O. coarctata*). Interestingly, species- and tissue-specific switches in the expression of these splice variants occur under salt stress, indicating that IR may contribute to the differential ability of *Oryza* NHX1 homologs to maintain ion-homeostasis under salt stress. IR in cold-responsive *OsCYP19-4* transcripts provides another example of a functional effect of AS [78]. The alternative transcripts OsCYP19-4.2/3 are truncated, but their interaction with AtRCN1 (regulator of auxin signaling in guard cells) indicates their role in cold acclimation by influencing stomatal physiology. Besides, most of these truncated isoforms show different subcellular localization under cold stress, indicating their organelle-specific roles [78].

From these studies, it is obvious that intron-retaining mRNAs are pivotal for regulating normal plant physiology. Therefore, IR should no longer be perceived as a selfish burden on the nucleus that only reduces gene expression with no functional significance in plant biology. In nutshell, the IR can be a robust mechanism to regulate gene expression with potential functional implications for rice stress tolerance.

##### Tissue- and/or Developmental Stage-Specific Expression of Transcript Variants

Consistent with the results from genome-wide studies, the expression of abiotic stress-induced individual mRNA variants of several regulatory and structural genes is development- or tissue-specific [73,79,83,99,101,102], while the variants from other genes show differential expression in various tissues and/or at developmental stages [77,81,96,102,103,104,105,106,107,108]. The spatial expression of splice variants has a potential significance for rice stress tolerance. For example, high expression of the functional form of *OsHKT1;4* in young rice sheaths causes their loading with Na^+^ ions to protect the young photosynthetic leaf blades from Na^+^ toxicity, while the lower levels of the functional form (due to higher expression of truncated forms *OsHKT1;4-SV1/SV2*) in the older sheaths let Na^+^ ions go through to the older blades for storage without harming the plant [73]. The expression of light-regulated AS transcripts of rice *pseudo-histidine phosphotransfer protein 3* (*OsPHP3*) is differentially modulated by auxin in rice roots, causing high root-specific expression of *OsPHP3.2/3.3* isoforms [83]; indicating that auxin may mediate the effect of light signaling on root growth by differentially regulating the abundance of AS transcripts of *OsPHP3*. Similarly, the higher ratio of functional to nonfunctional AS forms of *OsPCS2* (*OsPCS2a/OsPCS2b*) in the cadmium-stressed shoot tissue of rice potentially reduces the cadmium toxicity in shoots [99]. Likewise, the AS variants of cold-responsive *OsFe-SOD* show differential expression at different developmental stages of rice, with the expression of *OsFe-SODb* being higher at the vegetative stage, while that of *OsFe-SODa* being higher at the flowering stage [96]. Altogether, these results support the notion that tissue-specific AS events may play key roles in fine-tuning molecular and physiological processes in response to abiotic stress by regulating the relative abundance and function of the encoded proteins. They also signify that AS events may be important for mounting response to stress at particular developmental stages.

##### Stress and Non-Stress Roles of Alternative Transcripts

A transcript variant produced from a particular regulatory gene may be responsive to abiotic stress, whereas its other variant(s) may not be stress-responsive, but could be involved in other processes instead. For example, *OsRad9*, involved in the cell cycle check-point signaling pathway, generates transcript variants of *OsRad9.1* and *OsRad9.2*. OsRad9.1 is the full length and main cell cycle checkpoint protein involved in rice responses to different abiotic stresses; while the shorter OsRad9.2, having lost nine phosphorylation sites at its C-terminal, is highly expressed in pollen [79]. Since phosphorylation serves as a key modification in signaling pathways, the loss of phosphorylation sites in the shorter variant may have altered its function, suggesting that AS may expand the repertoire of functions of *OsRad9*—the usual role in the repair of DNA damage and a different role in pollen development. Similarly, the nucleolin gene, *OsNUC1*, generates two isoforms, the longer *OsNUC1-L* is involved in the root growth, and the shorter *OsNUC1-S* with no N-terminus is involved in salt tolerance as determined by the phenotypes of the *OsNUC1-L*- and *OsNUC1-S*-transgenic plants, respectively. It is proposed that the deletion of the N-terminal makes the shorter isoform function as an mRNA stabilizer for the other salt-tolerant genes under salt stress [80]. Similarly, *OsMAPK5* produces longer abiotic stress-induced *OsMAPK5a* with intact kinase activity and shorter *OsMAPKb* lacking this activity, suggesting AS regulation of this gene in response to stress [109].

##### Regulation of Subcellular Localization of Transcript Variants

The decision of whether an RNA is translated, modified, preserved, or degraded, is dictated by the intracellular location of the RNA, which in turn determines the biological function of the RNA [110]. Subcellular targeting can, therefore, have a significant influence on the regulatory and biochemical potential of the translated protein. AS influences the localization of spliced variants of some abiotic stress-associated genes of rice, thus regulating the function of their translation products. The rice senescence-related *ONAC054* mRNA produces *ONAC054α* which encodes an endoplasmic reticulum (ER) membrane-bound protein during normal conditions; however, the stress hormone abscisic acid (ABA) induces AS of *ONAC054* transcript to generate the truncated transmembrane domain (TMD)-less *ONAC054β* [82]. ONAC054β relocates from ER to nucleus, where it activates ABA signaling and senescence-related genes. Similarly, the rice ER stress regulator OsbZIP74 is encoded with an intact TMD under non-stress conditions; however, heat and ER stresses cause this domain to be spliced out from the mRNA of *OsbZIP74*, and the protein from this splice isoform is then relocated to the nucleus to promote the UPR-related gene expression, thus mitigating the heat and ER stress by enhancing the protein folding [111]. These results imply that the activity of TMD-containing transcription factors depends on their nuclear localization and TMD cleavage, which are in turn regulated by AS. In another case, alternative splice variants of a kinase gene, *OsBWMK1*, also exhibit differential subcellular localization under oxidative stress, with the larger isoform (OsBWMK1L) imported to the nucleus, but the localization of the smaller and medium variants (lacking the N-terminal sequence present in a larger variant) remaining the same [81]. Notably, salt and oxidative stresses up-regulate the expression of shorter and medium isoforms, but the expression of longer isoform does not change; instead, it responds to oxidative stress at the AS level by relocating to the nucleus and possibly activating the stress-responsive gene expression, indicating the functional significance of this AS event. It implies that OsBWMK1 isoforms act through distinct regulatory mechanisms in response to abiotic stress. Therefore, AS-mediated nuclear import of different proteins occurs via distinct mechanisms—in some cases, it may be a cause of cleavage of a sequence from the imported protein, and in other cases, it may cause retention of a sequence needed for protein import.

##### AS in UTRs of Pre-mRNAs

Besides the introns and exons in the coding region, AS also occurs in UTRs of pre-mRNAs [41]. One of the AS mechanisms used by most eukaryotes to increase transcript diversity of a gene is the use of alternative first exons by alternative promoter usage. In other words, AS occurring upstream of the first exon of pre-mRNA can result in the usage of alternative promoters by the transcript variants [112]. Thus, such transcript variants differ only in their 5′-UTRs. This mechanism substantiates the results obtained by Koo et al. [81], as discussed above. The differential expression of the three *OsBWMK1* transcript variants under stress results from AS and the alternative promoter usage [101]. The constitutive expression of larger isoform (*OsBWMK1L*) is due to the use of promoter-containing TATA-rich region, while the stress-induced expression of smaller isoform (*OsBWMK1S*) is due to the use of alternative promoter harboring several stress-responsive *cis*-elements. In a similar study, the transcript variants of *OsPHP3*, produced via alternative promoter usage, show differential expression in response to a light signal, with *OsPHP3.1* being the major transcript variant in the light-grown seedlings and *OsPHP3.2/3.3* predominating in the etiolated seedlings [83]. Notably, the analysis of the upstream sequence of *OsPHP3.2/3.3* pre-mRNA, which is spliced out in the case of *OsPHP3.1*, showed the presence of several light-related *cis*-elements, including nine activating sequence factor-2 (ASF2)-binding sequences, which were suggested to be highly activated under darkness. Thus, the alternative promoter usage for the generation of transcript isoforms provides an extra route to creating novel regulatory opportunities under stressful conditions. 

AS in 5′-UTRs can influence the efficiency of gene expression by generating transcript variants with altered uORFs [41]. It is reported that a uORF can either induce mRNA degradation via NMD or repress the translation of the main downstream ORF [41]. In the case of rice, the functional relevance of a uORF within the 5′-UTR of *Nitrogen Limitation Adaptation 1* (*OsNLA1*) has been demonstrated for the regulation of phosphate acquisition in roots [84]. This gene produces AS variants having long (*OsNLA1.1*) and short (*OsNLA1.2/1.3*) 5′-UTRs, with only *OsNLA1.1* containing a uORF of 30 amino acids. Among the three isoforms, the longer uORF-containing isoform is the most abundant and exhibits the highest expression in response to high phosphate stress in roots and shoots. Of note, unlike its homolog in Arabidopsis, *OsNLA1* is not regulated by the phosphate starvation-induced miR827 [113]. Instead, the uORF and other promoter elements were found to be cooperatively regulating the expression of *OsNLA1* for controlling the phosphate uptake and translocation [84]. Importantly, at different phosphate concentrations, uORF-mediated regulation of *OsNLA1* expression might occur through mechanisms, such as ribosome stalling, to induce the expression of *NLA1.1* at high and repress its expression at low phosphate concentration [114]. Hence, AS in the 5′-UTR plays a key role in phosphate stress tolerance by retaining a regulatory uORF in the most abundant isoform *OsNLA1.1* and substituting for the miRNA-mediated regulation. AS in 3′-UTRs can also produce variants with different stress-responsive roles. Rice *Nutrition Response and Root growth* (*NRR*) pre-mRNA undergoes AS in the 3′-UTR to produce a longer *NRRa* and a shorter *NRRb* transcript, which show varied expression patterns in roots and shoots under macronutrient deficiency, with the larger isoform playing a more crucial role in root growth [85]. Since the silencing of both isoforms results in more improved root growth than when either of them was suppressed, it indicates that these isoforms may act in concert under nutrient stress to modulate the rice root architecture. Therefore, the generation of transcript isoforms via AS in 3′-UTR may provide a means to facilitate the synthesis of functionally related proteins that cooperatively mediate a certain biological response. Likewise, the transcript of *OsCPK17* produces five splice variants with most of them down-regulated immediately under the cold stress; however, the expression of isoform *OsCPK17.5*, having no 5′-UTR and different 3′-UTR as compared to the other isoforms, changes considerably only after 72 h of stress, indicating that this isoform may be involved in cold tolerance during prolonged stress which is controlled by AS [108].

AS in the UTRs of structural genes also has important implications for rice abiotic stress tolerance. The amylose content in rice grains is temperature sensitive and depends on the 5′ splice sites of intron present in the leader sequence of granule bound starch synthase (GBSS)-encoding *waxy* gene [86]. Differential temperature-dependent utilization of leader intron splice sites results in the generation of GBSS transcripts with dissimilar 5′-UTR sequences. A splice site used at lower temperature causes a large deletion in the 5′-UTR sequence and also generates a uORF, which does not prevent the accumulation of mature GBSS mRNA. However, the splice site used at higher temperatures causes the production of a substantial amount of incompletely processed mRNA and also generates a uORF, which selectively degrades GBSS transcript [86]. The importance of UTRs in rice salt tolerance is presented by AS in the 3′-UTR of *OsNHX1*, which has three transcript variants—one of which has a truncated C-terminal end with a deletion of 150 bp in the 3′-UTR. Transgenic rice plants overexpressing the transcripts with intact 5′- and 3′-UTRs exhibit higher salt tolerance—due to significantly more K^+^ level and chlorophyll-retention at seeding stage and more spikelet fertility and yield at the reproductive stage—than those expressing the 3′-UTR-truncated transcript [87]. Because many of the rice stress-related genes undergo AS, the study underscores the importance of using full-length transcripts for generating more stress-tolerant transgenic plants.

##### AS and ABA-Mediated Responses

Phytohormone ABA, which is involved in the adaptive abiotic stress responses by controlling the expression of various stress-responsive genes, plays a crucial role in the AS-mediated regulation of rice abiotic stress responses. AS can target the components of ABA signaling to fine-tune the stress responses. For example, the transcript variants of *OsABI5* perform distinct functions in ABA signaling by differently interacting with ABA signaling protein OsVP1 and distinctly binding the stress-responsive ABA-related G-box element [115]. ABA also regulates key cellular processes in rice by controlling the AS of specific genes. ABA-mediated AS in the mRNA of ER-resident ONAC054 produces a transcript variant (*ONAC054β*) that is translocated to the nucleus for activating the genes related to senescence and ABA signaling [82]. Moreover, the transcript isoforms of SUMOylation-related gene *SUMO-conjugating enzyme 1a* (*OsSCE1a*) are differentially expressed in response to ABA [116], indicating that AS may fine-tune the regulatory effect of ABA on SUMOylation machinery. Further, although the functional transcript variant of *OsIM* (*OsIM1*) is induced by ABA, the high ABA concentrations repress it, indicating a potential feedback inhibition, and hence, the involvement of *OsIM1* in the ABA-biosynthesis [74]. The study also suggests that because ABA is the end-product of the carotenoid biosynthesis pathway, *OsIM1* can be involved in rice response to ABA by promoting carotenoid biosynthesis. Similarly, *OsMAPK5* has two splice isoforms, *OsMAPK5a* and *OsMAPK5b.* In contrast to *OsMAPK5b*, *OsMAPK5a* has kinase activity and is ABA-inducible. The *OsMAPK5a*-transgenic plants show increased drought, salt, and cold tolerance [109]. Along similar lines, alternatively spliced variants of *OsGATA23* are differentially regulated in salt-tolerant and sensitive rice genotypes in response to ABA and other abiotic stresses [107]. Collectively, these studies indicate that AS may adjust the effect of ABA on rice abiotic stress responses and that ABA mediates the post-transcriptional regulation of rice stress responses. In fact, some splicing regulators of rice (described below) show altered response to ABA [117,118,119]), implying that ABA may be involved in the control of rice abiotic stress responses by these splicing factors.

##### Regulation of AS by Abiotic Stress-Responsive Genes

In addition to the above-mentioned studies, other studies on rice abiotic stress-responsive genes have revealed that they are not themselves alternatively spliced but influence the stress tolerance of rice by regulating the splicing of other genes. For example, the rice immunophilin family gene *OsFKBP20-1b* participates in drought and salt stress responses by positively affecting transcription and pre-mRNA splicing of abiotic stress-related genes. OsFKBP20-1b-mediates this splicing by maintaining the protein stability of splicing factor OsSR45, and both of them work together during abiotic stresses to regulate the RNA metabolism [120].

#### 2.2.2. Circular RNAs

Circular RNAs (circRNAs), which are produced through back-splicing in pre-mRNA, play important roles in different plant biological processes and can regulate gene expression through modulating AS and sequestering miRNAs [121]. The functional role of these endogenous noncoding RNAs in rice stress responses is not fully explored. A genome-wide study has revealed the potential role of circRNAs in response to phosphate-starvation stress in rice [122]. The study found differential expression of 27 exoniccircRNAs under phosphate-deficient conditions, with six being significantly up-regulated and 21 down-regulated. Further, the expression of some of these circRNAs was correlated with that of their parent genes, implying their coregulation under phosphate-starvation stress. A recently developed comprehensive database for crop abiotic stress-responsive circRNAs hosts 63,048 rice circRNAs responsive to drought, salt, cold and other stresses, including their thorough details [123]. This database can be used to gain critical insights into the role of circRNAs in rice abiotic stress tolerance. Another database, called AsmiR (http://forestry.fafu.edu.cn/bioinfor/db/ASmiR, accessed on 20 February 2020), identifies the miRNA alternatively targets spliced transcripts and circRNAs in several plants, including rice [124].

Although circRNAs are regarded as noncoding, recent studies on mammals and plants provide evidence for certain protein-coding circRNAs [125,126].

#### 2.2.3. AS and Splicing Factors

AS is precisely regulated by a distinct group of RNA binding proteins, known as *trans*-splicing factors, which bind to specific RNA sequence signals called *cis*-splicing elements. Among these factors are SR proteins, which play a crucial role in the assembly and recruitment of spliceosome to splice sites by interacting with splicing elements and other splicing factors [20,21]. Plants have a higher number of *SR* genes than animals [127], perhaps as a way of building the capacity to respond to wide-ranging unavoidable environmental cues. SR proteins are closely involved in rice abiotic stress responses. For example, analysis of rice *sr* mutants has revealed that SR splicing factors have a regulatory role in maintaining the nutrient homeostasis in rice; with RS29 for Mn-, RS33 for Zn-, and many SR proteins (e.g., SR40, SCL57, SCL25) being essential for P-homeostasis in rice shoots [11]. In particular, the P remobilization from old to young leaves is significantly reduced in *sr40* and *scl57* mutants, suggesting that these two proteins could be involved in phloem transport of phosphate by positively regulating the expression of phosphate mobilizing transporters such as PHT. Another splicing factor, CWC25, is essential for spliceosome activity by modulating its structure at the first transesterification reaction [119]. The functional analysis of *CWC25* has revealed another example of the importance of AS in rice abiotic stress tolerance. The *cwc25*-knockout mutants exhibit reduced seed germination and shorter seedling roots as compared to wild-type under ABA and salt stress [119]. The spliceosome component, Ski-interacting protein (OsSKIPa), regulates cell viability and growth of rice under ABA, drought, and salt stress [117]. *OsSKIPa*-transgenic rice plants exhibit improved drought tolerance at seedling and reproductive stages, due to the high potential of scavenging the ROS and up-regulation of crucial stress-responsive genes, such as *SNAC1*, *CBF2*, *PP2C*, and *RD22*. OsSKIPa complements the lethal phenotype of yeast PRP45 (a splicing factor) mutant, indicating that OsSKIPa also functions as a splicing factor [117]. Functional analysis of yet another spliceosome-associated protein, DEAD-box RNA Helicase42 (OsRH42), has revealed its role in regulating effective pre-mRNA splicing under cold stress. In the cold-stressed *OsRH42*-knockdown and -overexpression plants, genome-wide AS, as well as AS of a subset of cold-responsive genes (e.g., *ALKALINE INVERTASE6* and *DST COACTIVATOR1*) is severely affected, resulting in retarded growth and cold-sensitivity [128]. The availability of single and multiple mutants of rice *SR* genes that were generated using the CRISPR/Cas9 technology should expedite the functional analyses of rice SR proteins in biotic and abiotic stress responses [129].

SR proteins being the master-regulators of AS, implies that they could themselves be tightly regulated. In fact, the *SR* transcripts undergo AS under ABA, NaCl, heat, and cold stresses, with their alternative transcripts exhibiting differential expression in three different rice ecotypes under these stresses [118]. Besides, most of their isoforms harbor PTC leading to the truncated products, which might act as an extra layer of regulation for the expression of their target genes under stress.

Collectively, these examples illustrate that splicing factors and spliceosome-associated components can be controlled by various abiotic stresses and can, in turn, regulate the rice responses to these stresses.

## 3. Biotic Stress

### 3.1. AS in Biotic Stress-Responsive Genes

As compared to abiotic stresses, relatively less progress has been made in deciphering the role of AS in rice biotic stress responses. However, the AS in biotic stress-responsive genes of rice generally acts through similar mechanisms as illustrated for the abiotic stress-related genes (Figure 1; Table S1).

#### 3.1.1. AS in Pre-mRNAs of Resistance (R) Genes

R proteins play a vital role in activating the host defense system by directly or indirectly interacting with virulence factors produced by the pathogens [130]. The most common class of R proteins in plants is composed of a central nucleotide-binding site (NBS) region and a C-terminal leucine-rich repeat (LRR) region. The expression of many plant *R* genes is regulated at the AS level in response to pathogen attack, underscoring the importance of this regulatory mechanism in plant defense [131]. However, in rice, only a handful of *R* genes are reported to be alternatively spliced and play a role in pathogen infection. Two of these genes are involved in blast disease-causing *Magnaporthe oryzae* defense response. For example, the *Pi-ta* gene, which confers resistance to blast, undergoes AS in a blast-resistant rice cultivar to produce a variety of transcript isoforms having truncated UTRs, retained introns, or extra C-terminal domains, due to the presence of new introns and novel splice sites [88]. Notably, among the different proteins produced from splice variants, the ones with the extra C-terminal domain (TRX domain) show the highest expression in the blast-resistant cultivar, indicating that the extra domain-containing isoforms can regulate the Pi-ta protein activity and may reflect a strategy evolved in the resistant rice variety to mount a better response to blast fungus. It also suggests that this increased transcript diversity can be an effective way by which rice R proteins recognize AVR-Pita variants in blast fungi populations and fortifies the R factors in R-AVR warfare. Similarly, another *R* gene, *RGA5*, produces two splice variants *RGA5-A* and *RGA5-B* [89]. Due to the retention of 3rd intron and frameshift in *RGA5-B* transcript, only *RGA5-A* confers resistance to *M. oryzae* effectors AVR1-CO39 and AVR-Pia. However, because *RGA5-B* encodes almost a full-length protein with a different C-terminal sequence and because it also interacts with AVR1-CO39 fungal effector, it may modulate the RGA5-effector interaction and the response to fungal infection. More specifically, the transgenic rice lines expressing the *RGA5-B* are fully susceptible to the fungal infection [89], indicating this isoform may act as a negative regulator of RGA5-A, which remains to be determined. The other two *R* genes are involved in response to destructive bacterial blight caused by *Xanthomonas oryzae* pv. *Oryzae (Xoo).* Rice *Xa10* is an executer *R* gene that provides resistance to *Xoo*. Transcripts of two *Xa10*-like genes (*Xa10-Ni* and *Xa23-Ni*) that encode ER membrane-localized proteins are induced in response to *Xoo* infection and effectively provide resistance to this infection [90]. *Xa23-Ni* transcript produces three alternative transcripts, out of which only the one with an IR event specifically confers tolerance to bacterial blight in the transgenic rice plants, possibly by depleting ER Ca^2+^ and triggering cell death, indicating a functional significance of this IR event. It also indicates that the other two isoforms may exist to regulate the level of functional isoform during infection. In yet another *R* gene *Non-expresser of PR3* (*OsNPR3*), the three transcript variants produced are responsive to *Xoo;* however, only *OsNPR3.3* is induced specifically in a resistant genotype, indicating that only this variant can play a role in the *Xoo* resistance signaling pathway [132]. The other splice variants are down-regulated during *Xoo* infection, suggesting that they may fine-tune the expression of OsNPR3 during infection. Although these studies indicate that AS in *R* genes provide some reinforcement in the disease resistance of rice, more efforts are needed to understand the biological roles and functional significance of AS in *R* genes for rice immunity.

#### 3.1.2. AS in Other Biotic Stress-Related Genes

##### Functional Significance of AS in Rice Immunity

In addition to the *R* genes, AS events in other biotic stress-related genes of rice also play key roles in immunity. All of these genes are regulatory in nature; involved either in transcription or signaling pathways associated with rice biotic stress responses. The functional significance of AS in some of these genes has been well illustrated, whereby the truncated alternative transcripts have provided vital insights into regulating rice biotic stress responses. For example, the canonical isoform may function as a negative regulator, while the alternative spliced form can be a positive regulator of rice disease resistance. The AS of *OsWRKY62* and *OsWRKY76* transcription factors, which cooperatively repress the rice defense to *M. oryzae* and *Xoo*, are altered during pathogen infection [91]. The alternatively spliced truncated transcripts *OsWRKY62.2* and *OsWRKY76.2* function to enhance the rice resistance to pathogen infection by decreasing the interaction between the canonical isoforms and greatly impairing their transcriptional activities. Moreover, the truncated isoforms exhibit diminished repressor activity as compared to the full-length proteins, and are highly up-regulated in disease-resistant *OsWRKY62.1/76.1*-knockout lines. Therefore, the generation of truncated WRKY variants causes the dominant-negative feedback regulation to contain the repressor activities of full-length forms, enabling the activation of WRKY-mediated defense against pathogens [91]. The negative regulatory role of *OsWRKY62.1* in rice innate immunity is also reported by Peng et al. [133]; however, the role of its alternative form *OsWRKY62.2* (in this case, however, produced by alternative first exon usage) is reported to not affect the rice resistance against *Xoo*, suggesting that different AS types can have profound consequences on stress tolerance. Similarly, another example of dominant-negative regulation via the production of truncated spliced form is provided by LAMMER kinase gene *OsDR11* [92]. The two alternative variants, longer *OsDR11L*, and shorter *OsDR11S*, play a contrasting role in rice defense against *Xoo*, with the larger (having kinase activity) and smaller isoforms suppressing and promoting the rice disease resistance, respectively. The negative regulatory effect of truncated isoform on the larger isoform is because the former inhibits the latter at both transcriptional and kinase levels, which leads to rice *Xoo*-resistance via releasing the OsDR11-L-mediated suppression of jasmonate (JA) signaling. Moreover, *OsDR11* contributes to a *Xoo*-responsive QTL operating through *OsDR11S* inhibition of *OsDR11L* [92]. In another case, alternative transcripts play the same role in rice pathogen resistance by elevating the JA levels, but in different ways and perhaps through different signaling pathways. While the *OsbZIP81.1* directly targets the genes (*OsLOX5*, *OsHI-LOX*, *OsAOC*, and *OsPIOX*) in JA signaling and biosynthesis pathway, its truncated alternative transcript *OsbZIP81.2* may regulate pathogenesis-related genes *OsPR10a* and *RSOsPR10* for adjusting the JA levels during infection [93].

##### Modulation of Subcellular Localization of Splice Variants

AS can also trigger effective plant defense responses against pathogens by influencing the regulatory properties of alternative translation products via modulating their subcellular localization. For example, AS causes the large splice variant of signal transduction gene *OsBWMK1* to translocate from cytoplasm to nucleus in response to defense-related signals, where it may interact with downstream target molecules to activate the desired responses [81]. Since the other two variants have their N-terminal regions truncated, the larger variant with intact N-terminal can phosphorylate itself and the other substrates, which allows its translocation to the nucleus [81]. Similarly, splicing of transcription factor *OsbZIP74* mRNA is associated with its translocation from ER to the nucleus for potentially activating systemic acquired resistance in response to a key defense-related hormone salicylic acid [111].

##### AS-Mediated Inverse Regulation of Rice Immunity and Abiotic Stress Tolerance

Some AS variants inversely regulate pathogen resistance and abiotic stress tolerance in rice. For instance, although both alternative transcripts of *OsMAPK5* are induced by blast fungus, only the larger isoform *OsMAPK5a* is induced by abiotic stresses. Moreover, *OsMAPK5a*-knockdown rice lines showed enhanced resistance to fungal and bacterial pathogens, whereas the same lines exhibit significantly reduced tolerance to drought, salt, and cold stresses [109], indicating that OsMAPK5a negatively and positively regulates broad-spectrum host resistance and abiotic stress tolerance, respectively. Likewise, the expression of the large isoform of *OsBWMK1* is not changed by NaCl, H_2_O_2_, or *M. grisea* elicitor [81]; however, it is highly induced by different *Xoo* strains and also enhances disease resistance against these bacterial strains in transgenic rice [134].

##### Impact of Sequence Variations in Biotic Stress-Related Genes on Splicing Events

Apart from these mechanisms, some sequence variations in biotic stress-related genes alter splicing events that may be associated with rice immunity. For example, *OsCUL3a* negatively regulates rice immunity to *M. oryzae* and *Xoo* by promoting degradation of OsNPR1, a master regulator of cell death in rice [135]. In pathogen-resistant *oscul3a* mutants, the mutation causes incorrect splicing, due to the disruption of the intron splice site in *OsCUL3a* pre-mRNA, leading to frameshift and introduction of PTC. Thus, splicing plays a vital role in the disease resistance of the mutants by facilitating the over-accumulation of a positive regulator of cell death OsNPR1, leading to the PCD and activation of immunity to restrain the pathogen infection. Similarly, *Bsr-k1* (Broad-spectrum resistance Kitaake-1) negatively regulates rice broad-spectrum disease resistance to *M. oryzae* and *Xoo* by promoting the mRNA degradation of multiple defense-related phenylalanine ammonia lyase genes (*OsPAL1-7*), which are involved in the phenylpropanoid pathway [136]. The mutation in broad-spectrum disease-resistant *bsr-k1* plants causes incorrect splicing by disrupting the 3′-splice site in the 8^th^ intron, which produces a new splice variant with a PTC, due to the retention of a partial intron sequence. This leads to high pathogen-resistance, due to the diminished turnover of *OsPAL1-7* mRNAs, causing enhanced accumulation of secondary metabolite lignin [136]. In another study, an SNP (C/T) in rice *wall-associated receptor kinase 91* (*OsWAK91*) gene, localized in a sheath blight resistance QTL region, differentiates sheath blight-susceptible and resistant rice varieties [137]. It was shown that this SNP is strongly correlated with genotype-specific genetic responses. For example, the SNP has more consequences on gene function and structure with increased splicing in resistant than susceptible genotype under biotic stress, which also might be associated with disease resistance.

##### Modulation of the Level of Functional Transcripts

In the case of rice protein phosphatase 2C gene *OsBIPP2C2*, AS is required to produce the functional protein OsBIPP2C2a [94]. Under normal growth conditions, all three alternative variants exist, whereas *OsBIPP2C2a* transcript greatly accumulates in response to defense-signal benzothiadiazole and blast fungus *M. grisea*, thus enabling rapid production of OsBIPP2C2 protein in response to fungal infection. Similarly, *OsPELOTA* has three alternative transcripts, among which only the full-length functional protein-coding variant is capable of conferring resistance against bacterial leaf blight [95], while the other two variants with unspliced introns and PTCs can be translated into truncated proteins to control the amount of functional OsPELOTA protein.

##### Splicing Factors

Studies concerning the biotic stress-induced AS in pre-mRNAs of splicing factor genes or the involvement of splicing factors in regulating rice responses to biotic stress are lacking. To the best of our knowledge, only one splicing factor gene, *SPL5* (*Spotted leaf5*), has been reported to negatively regulate rice resistance to blast and bacterial blight diseases [138]. The *SPL5* mutation was later found to activate multiple genes involved in programmed cell death, defense responses, and serotonin biosynthesis [139,140], which might promote rice resistance to bacterial and fungal pathogens.

##### Role of Noncoding RNA in AS for Rice Immunity

Besides the protein-coding genes, non-protein-coding RNAs also play critical roles in regulating rice immunity against different pathogens [141]. However, studies on AS in miRNA genes, the effect of AS on miRNA-mediated gene regulation, or the involvement of miRNAs in regulating AS of other genes under biotic and abiotic stresses also lack rice. The interaction between AS and miRNA targeting is illustrated in the case of Arabidopsis and rice [29,31,124,142]. In rice, only two studies exist so far showing the involvement of noncoding RNA in AS under biotic stress. Existence of a regulatory mechanism that potentially integrates miRNA function and mRNA processing has been illustrated in rice immunity against blast fungus [30]. The study showed that osa-miR7695 provides pathogen resistance by selectively cleaving and controlling the accumulation of only the shortest alternative spliced form of *Natural resistance-associated macrophage protein 6* (*OsNramp6*). Since the miRNA-target site is present only in the 3′-UTR of *OsNramp6.8* transcript, other alternative variants are not subject to this cleavage because of their shorter 3′-UTRs. Besides, *OsNramp6.8* plays a negative role in rice immunity because osa-miR7695-overexpressing plants show enhanced blast resistance. In a subsequent study, however, *OsNramp6.1* was also involved in rice immunity as its knock-out lines exhibited higher blast resistance [143]. The study also showed that *OsNramp6.1*/*6.8* are involved in iron and manganese transport. The expression of *OsNramp6.1* and *OsNramp6.8* alternative transcripts was shown to be up-regulated up to 24 h in pathogen-inoculated rice leaves and down-regulated thereafter. The initial accumulation of transcripts may indicate increased iron-uptake in competition with the pathogen; the decline after 24 h may indicate miRNA- (for *OsNramp6.8*) and other non-miRNA-mediated (for *OsNramp6.1*) feedback negative regulation for iron homeostasis.

Besides, a recent study has revealed the involvement of noncoding regulatory circRNAs in the interaction of rice with blast pathogen [144]. Expressed circRNAs were found to be much more diverse and abundant in blast-resistant than -susceptible rice genotype, especially during blast infection. The differential response of contrasting rice genotypes to blast fungus was ascribed to the differences in frequency of alternative back-splicing, the efficiency of AS inside circRNAs, and the use of complex splice sites. Moreover, circR5g05160 was found to enhance rice resistance to blast, and was predicted to target osa-miR168-5p and osa-miR2103 [144]. The circRNAs may, therefore, represent a new layer of regulation in rice responses to pathogens.

### 3.2. Transcriptomic Analysis of Biotic Stress-Induced AS in Rice

The importance of AS in rice responses to biotic stressors has also been revealed in a few transcriptomic studies. For example, gene expression analysis of specialized giant cells, that are specifically induced by root-knot nematode *Meloidogyne graminicola* in the rice root tissue, has revealed the presence of 16,063 alternatively spliced transcripts, which could provide useful insights into the rice-nematode interactions [145]. A comparative transcriptome profiling in durable resistant and susceptible rice genotypes for elucidating their early responses to *M. oryzae* has revealed that blast differentially regulates the components of rice defense signaling pathways, including the MAPK pathway. It was shown that some *MAPKKK* genes are modulated at the AS level only in resistant genotype, with some transcript variants of *MAPKKK.3* and *MAPKKK.1* genes up-regulated only in the resistant, but not in susceptible genotype, which could contribute to blast resistance of the former [146]. Similarly, AS in 18% of rice genes has been detected in response to *Spodoptera exigua* and *Lissorhoptrus oryzophilus* insect attack [147]. Importantly, some induced defense-related genes (e.g., genes encoding metallothionein-like protein type 2, nonspecific lipid transfer protein, aspartic proteinase precursor, BTH-induced protein phosphatase 1, etc.) produced alternative transcripts in insect-infested rice plants. In yet another transcriptomic study, alternatively spliced forms of some genes (e.g., *Os08g43334* encoding HSF-type DNA-binding domain-containing protein, *Os05g28960* encoding an acyltransferase) exhibit opposite expression patterns in response to *M. grisea* infection [148]. AS is an important stress-responsive mechanism that even pesticide application influences biotic stress tolerance through this regulatory process. In this connection, gene expression analysis in response to pesticide application have revealed the induction of 274 AS events in 270 genes of rice, which may play a potential role in rice biotic stress tolerance. Some important genes that were subjected to AS in pesticide-applied rice include *Os03g60430* (AP2 domain protein), *Os03g12620* (glycosyl hydrolases family 17), *Os11g32880* (DEAD-box ATP-dependent RNA helicase), etc. [149], indicating that defense mechanisms in pesticide-applied rice could be potentially mediated by AS by reprogramming various metabolic pathways.

Together, the biotic stress-responsive alternative transcripts identified in these studies reveal significant post-transcriptional responses of rice to pathogens and require further detailed analysis to understand the rice immune responses. Despite the above-mentioned studies on AS in rice biotic stress responses, the relevance of this regulatory mechanism is yet to be fully and critically explored in rice immunity.

### 3.3. AS in Rice Pathogens

**Figure 3:**
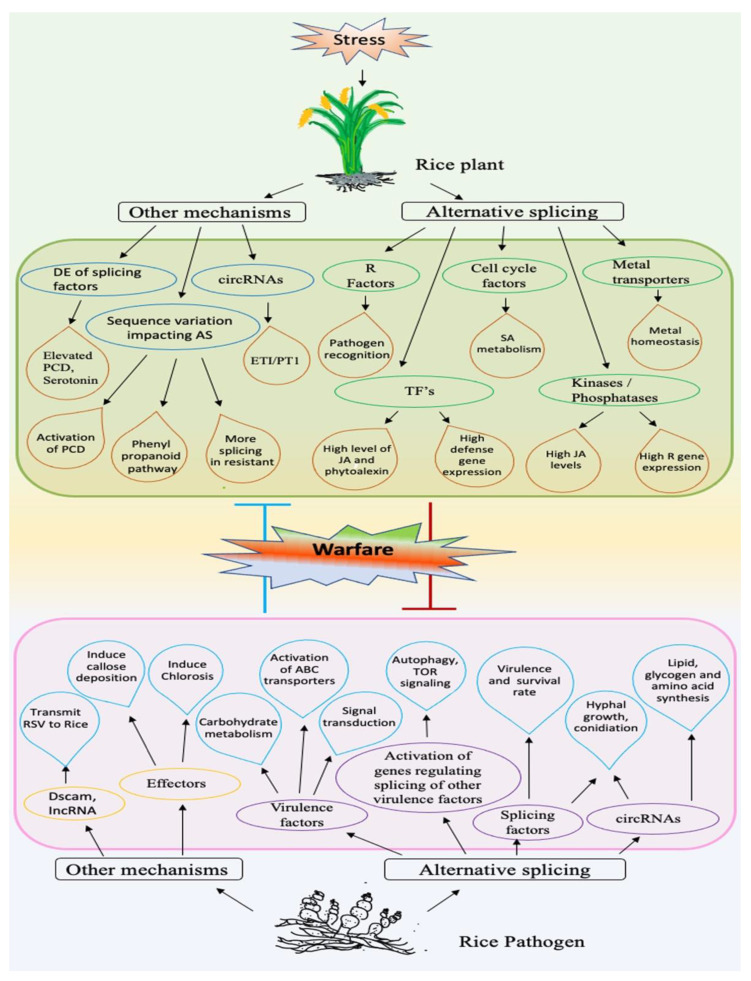
Participation of potential AS-mediated defense and offense processes in rice-pathogen interaction. Based on the published reports, the role of AS in the virulence of various pathogens and the potential AS-mediated processes that rice employs to counter stresses are shown. Besides the processes where genes are directly alternatively spliced, other mechanisms that regulate the expression of splicing factors and circRNAs, or the impact of sequence variations among different genotypes on AS, are also shown. AS, alternative splicing; CircRNAs, circular RNAs; DE, differential expression; ETI, effector-triggered immunity; JA, jasmonate; lncRNA, long noncoding RNA; PCD, programmed cell death; PTI, PAMP-triggered immunity; RSV, rice stripe virus; SA, salicylic acid; TAR, target of rapamycin; TFs, transcription factors.

Besides the elucidation of molecular defense mechanisms in rice, the understanding of mechanisms by which different pathogens adapt to various rice genotypes or infect rice is equally important to restrain the burden of biotic stress on rice for sustainable yield (Figure 3). Rice pathogens, such as bacteria, fungi, and insects, have evolved different strategies to subvert rice defense responses [150,151,152]. Although genome sequencing and transcriptomic analyses of various rice pathogens have provided a global view of disease-causing genes and have contributed to our understanding of the mechanisms by which these pathogens infect rice, yet the pathogen-responses to rice resistance mechanisms are not fully explored. The ubiquity of AS mechanisms in different vital biological pathways suggests that targeting this regulatory process in pathogens may be an effective means to control rice diseases. The molecular diversity in the biological processes of rice pathogens is known to involve AS. Therefore, we review the literature on the role of AS in regulating the growth, development, or virulence of different rice pathogens to gain insights into developing sustainable and environmentally friendly disease-control methods.

#### 3.3.1. Role of AS in Fungal Virulence and Growth

AS events occur in fungal virulence factors, which may be required for suppressing rice immunity and exploiting its metabolism to promote disease. For example, AS has been found to occur in 49.5% of genes (with IR as the most abundant event) of sheath blight fungus *Rhizoctonia solani* AG1IA during infection of rice, soybean, and corn [153]. Among these genes are some important pathogenesis-associated genes coding for ABC transporters, carbohydrate-active enzymes, signal transduction proteins, secreted proteins, and cysteine-rich proteins. Particularly, the *R. solani* candidate effector gene *AG1IA_03552* was alternatively spliced with intron-retention. Moreover, 12 fungal genes were commonly alternatively spliced during infection of all three hosts, including some up-regulated genes that were predicted to be pathogenicity-related and coding for cytochrome P450-domain-containing protein and glycoside hydrolase family 16 protein [153]. The high proportion of AS, especially in pathogenesis-associated genes, suggests that it could be an important gene regulatory process enabling *R. solani* AG1IA infection of rice. Similarly, 134 genes are potentially alternatively spliced in *M. grisea*, with most of the alternative transcripts generated through IR or differential 5′-splice site usage [154].

Besides, some other genes influence fungal virulence by regulating the splicing of genes necessary for pathogenicity. Arginine methyltransferase gene *MoHMT1* is essential for healthy vegetative growth, autophagosome formation, and full pathogenicity of *M. oryzae* [155]. *MoHMT1* regulates autophagy (vital for fungal development and pathogenicity) by methylation of a spliceosome component MoSNP1 which in turn remodels the AS process of several crucial autophagy-related (ATG) genes, especially *MoATG4* [155], indicating that accurate AS is crucial for development and autophagy in rice blast fungus. Likewise, the full virulence and development of *M. oryzae* are also modulated by RNA binding protein RBP35, which regulates the 3′-UTR length of alternative transcripts of several genes (encoding 14-3-3 protein, 40S ribosomal subunit S7, and aspartate semialdehyde dehydrogenase) essential for developmental and infection-related processes through modulation of TOR (target of rapamycin) signaling pathway [156].

Further, the characterization of circRNAs from *M. oryzae* mycelium and conidium has revealed their potential roles in the normal growth and virulence of the rice blast fungus [157]. Out of a total 8848 circRNAs identified, 2721 and 5840 were specific to conidia and mycelia, respectively, indicating the involvement of these circRNAs in regulating specific fungal developmental stages. Moreover, the parental genes of mycelial circRNAs were involved in metabolic processes related to normal fungal growth, while those of conidia circRNAs are involved in the biogenesis of lipids, glycogen, and amino acids to be used for the appressorium-mediated infection of the host [157].

#### 3.3.2. Role of AS in Virulence of Insect Pests

AS also regulates the normal growth, development, and virulence of different insect pests, which inflict damage to the rice crop. Among them, AS has been especially studied in brown planthopper (BPH), *Nilaparvata lugens*, because this phloem feeder insect pest imposes severe damage to rice—mainly during the reproductive stage [152]. The ‘hopper burn’ of rice caused by this insect pest often leads to wilting and subsequent plant death, thus devastating the rice yield or even causing the complete yield loss [158]. Studies suggest that AS may potentially help BPH to feed on rice plants, and possibly contribute to counter diverse defenses against BPH feeding that rice has evolved. Elucidation of responses of BPH to resistant rice has revealed that AS occurs in almost 18% of total protein-coding genes of BPH, with 3119 genes producing 5988 novel alternative transcripts [152]. Moreover, 915 fusion genes generated by *trans*-splicing were detected in the transcriptome of BPH maintained on resistant rice. Thus, alternative- and *trans*-splicing enhance the transcript diversity of BPH, possibly as a means to counter the rice immunity. Similarly, a secretome analysis has detected six important effectors from the salivary proteins of BPH, which may be important for BPH-rice interactions, and among the six, the Nl40 family (Nl40-1 and Nl40-2) produce 17 alternative splice variants, expanding the repertoire of effector proteins in BPH [159]. Through transient expression assay, Nl40 was demonstrated to cause chlorosis and callose deposition in *Nicotiana benthamiana* leaves [159]. Therefore, considering their effector properties and the AS, *Nl40* gene family members may contribute to complex BPH-rice interactions and enable BPH to rapidly adapt to rice. Consequently, these pathogenesis-associated factors of BPH can be targeted to block its virulence for sustainable pest management and rice yield.

#### 3.3.3. Role of AS in Insect Growth, Development and Fecundity

Besides its role in the BPH virulence, AS also plays an important part in insect development and fecundity. For example, sugar gustatory receptor gene *NlGr10* produces two alternative splice variants (*NlGr10a* and *NlGr10b*) by alternative first exon usage, which completely differ in their 5′-UTR sequences. The two alternative transcripts differently regulate BPH fecundity by binding distinct ligands and through participating in different signaling pathways; with NlGr10a promoting fecundity through AMPK and AKT-*Nl*Vg/*Nl*VgR in fat bodies and ovaries, while NlGr10b doing so through AMPK- and AKT-*Nl*Vg signaling pathway only in fat bodies [160]. *Nl*Vg and *Nl*VgR are regarded as the molecular markers of fecundity in BPH [161]. The knockdown of both isoforms severely affects the insect egg-laying and the egg hatching, with *NlGr10a*-knockdown insects being more impaired in these aspects [160]; thereby making AS of *NlGr10* a potential target for curbing the population of this serious sap-sucking pest of rice. Chitin plays a crucial role in insect growth and development by maintaining the structural integrity of various vital morphological and anatomical body parts [162]. For maintaining normal insect growth and development, the remodeling of chitin is necessary during the molting stage [163]. The chitin synthesis gene *CHS1* produces two transcript variants (*CHS1a* and *CHS1b* by alternative exon-19a and exon-19b), each in *N. lugens* and *Laodelphax striatellus* rice pests [164]. The *NlCHS1* splice variants differ in the tissue-specific expression, indicating their distinct roles in the tissue-specific chitin deposition. They also differ in their expression during molting, with *NlCHS1a* expressed after and *NlCHS1b* expressed before molting, indicating that the isoforms play different roles in the BPH development during the molting period. Moreover, knockdown of *NlCHS1* and its two alternative transcripts results in BPH insects with lethal phenotypes [164]. Similar results have been obtained with the *CHS1* gene (*SfCHS1*) of another serious insect pest of rice known as white-backed planthopper ‘*Sogatella furcifera*’ [163]. Together, these results suggest that *NlCHS1* and *SfCHS1* can be ideal targets for curbing the sap-sucking insect population through feeding corresponding dsRNAs by expressing them in plants.

Long noncoding RNAs (lncRNAs) play a crucial role in wide-ranging fundamental biological processes. In BPH, almost 20% of lncRNAs undergo AS [165], with the alternatively spliced isoforms of some lncRNA genes having different expression during different developmental stages (e.g., five out of 10 isoforms of *BPHLNC-unc241* gene are differentially expressed in adult and 3rd, 4th, 5th instar nymphs), implying their distinctive roles in BPH development.

Therefore, targeting different genetic elements involved in pathogen growth and development can be an effective mode of thwarting their population growth, and thus, lessening the biotic stress in rice.

#### 3.3.4. Role of AS in Vectoring Rice Pathogens

Besides itself being destructive to rice crop by phloem-feeding, the notorious *Laodelphax striatellus* (small brown planthopper, SBPH) further contributes to rice biotic stress and yield loss by vectoring a major crop pathogen RSV (rice stripe virus). This suggests that RSV infection of rice can be significantly curbed if the SBPH population is checked. The extensive AS in an insect gene encoding ‘Down syndrome cell adhesion molecule’ (Dscam) helps insects recognize various pathogens [166]. The Dscam of SBPH (LsDscam) also has a diverse protein isoform repertoire (71,400 isoforms) generated by AS, and it mediates the RSV entry into the cell and promotes virus infection of SBPH [167]. Notably, *LsDscam*-knockdown insects show diminished RSV burden [167], which suggests that specific alternative forms of LsDscam may promote the RSV infection of the SBPH vector, which in turn can horizontally transmit the virus to rice. Thus, suppressing the AS of *LsDscam* can potentially reduce the virus infection of the vector, and can ultimately lead to lesser biotic stress in rice. Likewise, lncRNAs can facilitate the successful infection of RSV in SPBH by potentially interacting with immune-related genes [168]. Although the study reports that 26.4% of lncRNA genes of SBPH are alternatively spliced, it does not provide evidence of the involvement of AS in RSV transmission in SBPH.

#### 3.3.5. Role of Splicing Factors in Growth, Development, and Virulence of Rice Pathogens

The important roles of a few splicing factors in the growth, development, and virulence of rice pathogens have been identified in recent genetic and transcriptomic studies. For example, the auxiliary splicing factor gene *MoGRP1* (encoding a glycine-rich protein) modulates growth, development, virulence, and stress responses in rice blast fungus [169]. MoGRP1 interacts with spliceosome components and plays a crucial role in modulating hyphal growth and conidiation by regulating the normal splicing of the corresponding genes, such as *MST7* and *MoRAD6*. Furthermore, besides their impaired mycelial growth, conidiation, and virulence, the *Mogrp1* mutant fungi are unable to cause lesions on rice leaves [169]. Similarly, the silencing of SR gene *NlSRp54* greatly decreases the survival of virulent BPH on resistant Mudgo rice, implicating an essential role of AS in the BPH-rice interaction and possibly in the rice resistance breakdown [158]. Another splicing factor ‘female determinant factor’ (NlFmd) modulates sex determination in BPH by regulating female-specific AS of the most conserved gene in sex determination cascade *doublesex* (*Nldsx*), and is, thus, important for female development [170]. Interestingly, *NlFmd* is itself alternatively spliced into female-specific *NlFmd-F* and sex nonspecific *NlFmd-C* isoforms, suggesting two layers of regulation on sex determination of BPH. Further, silencing of *NlFmd-F*, but not *NlFmd-C*, impairs the female-specific splicing of *Nldsx* and produces infertile females [170], which indicates that RNAi strategy can be used to develop sterile females for sustainable insect control, and thus, for the diminished biotic stress in rice.

## 4. Future Outlook for Research on AS in Rice Stress Responses

The critical evaluation of the studies presented in this article has ascertained the importance of AS in rice responses to environmental stresses and raised many novel questions (see below) that need to be addressed in the coming years.

1. How do stresses regulate AS in rice? Recent studies in plants indicate that much of the AS occurs co-transcriptionally [171,172,173], and that chromatin architecture (epigenetic state—histone modifications and DNA methylation, and nucleosome occupancy) [25,26,27,174] plays a key role in modulating AS. Analysis of AS in chromatin-bound RNAs in rice in response to different stresses should reveal the extent of stress-regulated co-transcriptional AS. By performing AS analysis and epigenetic analyses simultaneously in response to stresses, it should be possible to identify stress-induced AS events that are regulated by specific epigenetic changes.
2. The cross-talk between miRNA and AS pathways is poorly explored in rice abiotic stress responses. For example, it is not known how AS influences miRNA-mediated regulation of rice stress-responsive genes. The AS can have a major effect on miRNA-binding sites in the 3′-UTR of genes [41], and can also affect the process of miRNA excision from the primary-miRNA transcript to regulate the mature miRNA levels, and thus, the corresponding mRNAs in the cell [29].
3. AS is a critical component of stress priming-induced memory, which promotes the tolerance of plants to subsequent lethal stress [46]. Except for a preliminary report [175], it is not known whether the stress priming alters the choice of alternative splice sites in pre-mRNA transcripts of rice genes and whether it induces splicing memory, and should be explored.
4. The potential of CRISPR technology has been exploited in animals to modulate splicing for correcting the mutations associated with diseases [176]. However, in plants, especially rice, there is no report on improving any trait using this strategy. A recent study on Arabidopsis has developed an efficient base-editing tool for gene splicing [177]. This tool can be potentially used for elucidating the regulatory function of AS in plant responses to stress through generating the mutants for specific splice variants.
5. Genetic variability can profoundly influence the AS and abundance of alternative transcripts. The functional impact of genetic variations in shaping AS events with stress-adaptive consequences has also been revealed in plants. For example, microsatellites are involved in modulating AS of miRNA genes under different stress conditions [178], and ‘splicing QTLs’ (sQTLs) potentially regulate AS of various stress-responsive genes [179]. However, the significance of microsatellites and sQTLs in regulating AS for rice stress tolerance has yet to be determined.
6. The emergence of single-cell RNA-seq has revealed gene expression signatures, including AS, distinguishable at the cellular level, which is invisible in whole plant or organ RNA-seq. Isoform expression analysis at the single-cell level is crucial to gain deeper insights into AS biology. The specific stress responses emanating from AS in a particular cell-type of shoot or root tissues of rice have not been revealed yet and should be explored.

Addressing these questions will provide a comprehensive understanding of stresses-regulated AS in rice and open new avenues to develop climate-smart and disease-resistant rice varieties with improved yield potential.

## 5. Conclusions

Several global transcriptomic studies on rice in response to diverse environmental stresses have revealed that these stresses profoundly influence the AS of numerous pre-mRNAs—especially those encoding proteins involved in stress responses. However, the functions of most of the stress-induced splice variants are yet to be uncovered. Although the role of a handful of splice isoforms in stress tolerance has been defined, yet most studies are descriptive, and the functions of most stress-regulated splice variants are not known. Further, the mechanisms by which stresses alter splicing patterns are largely unknown. Thus, the functional characterization of key splice isoforms is critical for biotechnological applications towards enhancing rice stress tolerance. Since it is not practical to analyze the function of every stress-regulated splice isoform, it would be necessary to identify potentially important splice isoforms based on several criteria and test their role in stress tolerance. Elucidation of AS mechanisms of these splice isoforms under stress will require the identification of their splicing regulators and signaling pathways that transduce stress signals to splicing machinery.

## Data Availability

Not applicable.

## Supplementary Materials

The following are available online at https://www.mdpi.com/article/10.3390/biology10040309/s1, Table S1: Alternative splicing in different rice genes with functional significance for abiotic and biotic stress tolerance.
