# Supplementary material for: Stress-Induced Changes in Alternative Splicing Landscape in Rice: Functional Significance of Splice Isoforms in Stress Tolerance
Source: Biology (Basel). 2021 Apr 8;10(4):309. doi: 10.3390/biology10040309 (PMC8068108; doi:10.3390/biology10040309)
Supplement: Supplementary file 1 [file biology-10-00309-s001.pdf]

Table S1: Alternative splicing in different rice genes with functional significance for abiotic and biotic stress tolerance

Gene	Stress	Function	Type of alternative splicing	No. of isoforms	Functional significance (Mechanism)	Reference
**ABIOTIC STRESS**						
OsDREB2B	Drought, heat	TF regulates drought and heat stress-responsive gene expression	Frameshift and PTC due to exon-2	2	Controls the abundance of functional isoform *OsDREB2B2* under stress, which increases drought tolerance by enhancing the plant survival rate and relative water content.	[70]
myb7	Hypoxia	Stress-related TF	Intron-retention	2	Fine-tunes the amount of functional isoform under stress, which increases the expression of *myb*-related genes in hypoxic roots.	[71]
OsHSFA2d	Heat	TF regulates the expression of heat-shock proteins	Intron-retention	3	Fine-tunes the amount of functional isoform *OsHSFA2dI* under stress, which participates in the regulation of unfolded protein response.	[72]
OsHKT1;4	Salt	Na^+/K^+ transporter causes exclusion of Na^+ from photosynthetic tissues	Splicing instability caused by GC splicing donor splice site of intron-2	3	Salt-tolerant rice minimizes the Na^+ load in the young photosynthetic leaf blades by maintaining a very high ratio of functional *OsHKT1;4* isoform in the leaf sheaths over salt-sensitive rice.	[73]
OsIM	Salt	A homolog of alternative oxidase that functions as terminal oxidase in the alternative respiratory pathway	Incorrect splicing results in intron-retention	2	Maintains the high ratio of functional isoform *OsIM1* under salt, which may relieve salinity-induced oxidative stress.	[74]
OsPIF14	Cold	TF is involved in phytochrome signaling. It interacts with phytochromes	PTC due to intron-retention	2	Up-regulation of inactive isoform *OsPIF14β* at low temperature decreases the amount of functional protein and releases the repression of OsDREB1B to increase cold tolerance.	[75]

OsbZIP58	Heat	Endosperm-specific TF regulates the accumulation of storage material	PTC due to intron-retention	2	Up-regulation of *OsbZIP58β* at high temperature decreases the amount of functional protein and reduces the *trans*-activation of genes associated with the grain filling.	[76]
OsPIN1	Drought	Polar auxin transport	Intron-retention	2	The intron-retention event is highly up-regulated in deep roots of high RDR genotypes and is involved in modulation of deep rooting likely by facilitating the auxin transport.	[68]
OsMus81	Intense light, UV-C, γ-rays	Endonuclease involved in DNA repair	PTC due to splicing of a cryptic intron within exon 14	2	By interacting with different DNA repair proteins, the two isoforms (OsMus81α, OsMus81β) may repair the DNA damage through different repair mechanisms.	[77]
OsCYP19-4	Cold	Peptidyl prolyl cis/trans isomerase	Intron-retention and exon skipping	3	Because of their interaction with auxin signaling protein AtRCN1, the truncated isoforms OsCYP19-4.2 and OsCYP19-4.3 may modulate cold acclimation by influencing stomatal physiology .	[78]
OsRad9	Drought, salt, heavy metal, genotoxicity	Cell cycle check-point protein detects DNA structural abnormalities	Loss of exons	2	Causes functional divergence. OsRad9.1 is stress-responsive, while OsRad9.2 is involved in pollen development	[79]
OsNUC1	Salt	Pre-rRNA transcription and ribosome assembly	-	2	Causes functional divergence. OsNUC1-S is involved in salt tolerance, whereas OsNUC1-L in root growth.	[80]
OsBWMK1	Salt, H_2O_2	Kinase involved in signal transduction	Alternative first exon (Different 5'-UTRs)	3	Affects subcellular localization. The larger OsBWMK1L, but not the small and medium isoforms lacking the first exon, is translocated to the nucleus in response to oxidative stress for potentially activating gene expression.	[81]

ONAC054	ABA	TF associated with senescence	Alternative 3′ splice site selection	2	Affects subcellular localization. The truncated isoform ONAC054β translocates from ER to nucleus where it activates ABA signaling and senescence-related genes	[82]
OsPHP3	Light	Transfers phosphoryl group in the nucleus	Alternative promoter usage (Different 5'-UTRs)	3	*OsPHP3.1* with the shorter 5'-UTR predominant in the light-grown seedlings and *OsPHP3.2/3.3* with longer 5'-UTRs predominant in the etiolated seedlings	[83]
OsNLA1	Phosphate stress	Mediates ubiquitination	Alternative promoter usage (Different 5'-UTRs)	3	Only *OsNLA1.1* has a uORF in its 5'-UTR which substitutes the role of miR827 in regulating phosphate transport	[84]
NRR	Macronutrient deficiency	-	Different 3'-UTRs	2	*NRR1.1* with longer 3'-UTR plays more crucial role in root growth. *NRR1.1/1.2* may cooperatively modulate the rice root architecture under nutrient stress	[85]
GBSS	Heat	Synthesis of amylose in the endosperm	Differential usage of leader intron alternate splice sites in 5'-UTR	-	Splice sites used at lower and higher temperature cause the accumulation and degradation of *GBSS* mRNA, respectively	[86]
OsNHX1	Salt	Ion-homeostasis	Different 3'-UTRs	3	Transcripts with full-length 3'-UTR confer more salt tolerance than those with truncated 3'-UTR	[87]
**BIOTIC STRESS**						
Pi-ta	Blast	R gene recognizes pathogen AVR-Pita effector	Intron retention, Different 5'- and 3'-UTRs	3 in blast-susceptible, 11 in blast-resistant rice	The isoforms with the extra C-terminal TRX domain show the higher expression than full-length and truncated isoforms in the blast-resistant cultivar	[88]
RGA5	Blast	R gene recognizes pathogen AVR1-CO39 effector	Frameshift due to intron-retention	2	Only canonically spliced *RGA5-A* confers resistance to *M. oryzae* effectors AVR1-CO39 and AVR-Pia. Alternatively spliced *RGA5-B*-encoded protein also interacts with AVR1-CO39 fungal effector and may modulate the RGA5-effector interaction	[89]

Xa23-Ni	Bacterial blight	R gene recognizes pathogen transcription activator-like (TAL) effector	Intron-retention	3	The isoform with IR event confers tolerance to bacterial blight in the transgenic rice possibly by depleting ER Ca^{2+} and triggering cell death	[90]
OsWRKY62, *OsWRKY76*	Blast and bacterial blight	TFs involved in various signaling pathways	Intron-retention for *OsWRKY62*, splicing of a large intron in *OsWRKY76*	2 each	The truncated isoforms OsWRKY62.2 and OsWRKY76.2 have a dominant negative regulatory function. They enhance the rice pathogen resistance by impairing the transcriptional activities of full-length isoforms	[91]
OsDR11	Bacterial blight	LAMMER kinase	Frameshift and PTC due to the exclusion of exons 5, 6, and 7	2	The truncated isoform OsDR11S promotes rice blight-resistance by dominantly inhibiting OsDR11L both at transcriptional and kinase levels	[92]
OsbZIP81	Agrobacterium	TF	PTC due to retention of longer exon-2; different 3'-UTRs	2	Both full-length OsbZIP81.1 and truncated OsbZIP81.2 isoforms have the same function to adjust jasmonate levels during pathogen infection but in a different way and perhaps through different signaling pathways	[93]
OsBIPP2C2	Blast, Benzothiadiazol e	Balances protein dephosphorylation	PTC due to aberrant splicing in exon-3	2	Fine-tunes the amount of functional transcript *OsBIPP2C2a,* which functions in disease resistance by activating the defense-related genes	[94]
OsPELOTA	Bacterial blight	Regulates cell division cycle by influencing quality control of proteins	PTC due to intron-retention	3	Fine-tunes the amount of functional transcript *OsPELOTA1,* which functions in disease resistance by regulating salicylic acid metabolism genes	[95]
OsNramp6	Blast	Metal transporter	Different 3'-UTRs	8	Only the shortest isoform with the longest 3'-UTR is negatively regulated by osa-miR7695, while the other seven isoforms with shorter 3'-UTRs escape direct repression by this miRNA	[30]

TF – Transcription Factor, PTC – Premature Termination Codon, UTR – Untranslated Region
